# Serum acylcarnitines profile at ICU discharge to predict mid-term muscle outcomes: an exploratory study

**DOI:** 10.3389/fmed.2025.1622116

**Published:** 2025-10-31

**Authors:** Anne-Françoise Rousseau, Frédéric Farnir, Etienne Cavalier, Géraldine Luis, Isabelle Kellens, Bernard Lambermont, François Boemer

**Affiliations:** ^1^Intensive Care Department, University Hospital of Liège, University of Liège, Liège, Belgium; ^2^GIGA-Research, GIGA-I3 Thematic Unit, Inflammation and Enhanced Rehabilitation Laboratory (Intensive Care), University of Liège, Liège, Belgium; ^3^Biostatistics and Bioinformatics Applied to Veterinary Sciences, Faculty of Veterinary Medicine, University of Liège, Liège, Belgium; ^4^Clinical Chemistry Department, University Hospital of Liège, University of Liège, Liège, Belgium; ^5^Biochemical Genetics Lab, Department of Human Genetics, University Hospital of Liège, University of Liège, Liège, Belgium

**Keywords:** acylcarnitines, critical illness, survivors, muscle, outcomes

## Abstract

**Background:**

Alterations in acylcarnitines (AC) profile may reflect mitochondrial and metabolic disturbances after critical illness. This study investigated the association between AC profile at ICU discharge (ICUD) and muscle outcomes assessed 3 months (M3) later in survivors of a prolonged stay in ICU.

**Methods:**

Adults enrolled in our post-ICU clinic were included if they completed the ICUD assessment and attended the M3 consultation. Blood analysis was routinely performed at both time points, including AC profile. Muscle outcomes included urea/creatinine ratio, sarcopenia index, quadriceps and handgrip strengths, maximal inspiratory and expiratory pressures. Muscle health was defined arbitrarily as a composite of all these muscle parameters.

**Results:**

A total of 127 patients [age 63 (55–70) years] survived an ICU stay of 13 (8–33) days and were analysed. Free carnitine (C0) concentration was 44.4 (33–52.2) μmol/L. The total AC/C0 ratio (normal ≤ 0.4) was 0.37 (0.28–0.47). An AC/C0 ratio >0.4 was observed in 55/127 (43.3%). The short-chain and long-chain ACs reached, respectively, 1.2 (0.9–1.7) μmol/L and 0.9 (0.6–1.2) μmol/L. At M3, the urea/creatinine ratio and the sarcopenia index were, respectively, 38.3 (28.3–50.3) and 0.7 (0.6–0.9). Quadriceps strength was 2.9 (2.1–3.7) N/kg and handgrip strength was 25 (19–34) kg. In univariate analysis, none of the AC markers were associated with any of the muscle outcomes. A multifactorial linear model, including metabolic and AC markers, failed to predict M3 muscle health.

**Conclusion:**

In our exploratory cohort, AC profile as an isolated marker failed to predict post-ICU muscle weakness as assessed in daily practice.

## Background

Muscle weakness and secondary physical function impairment are common after a stay in intensive care unit (ICU). It is a predominant component of the post-intensive care syndrome (PICS) (1, 2). Muscle wasting, altered muscle composition, poor regeneration, abnormalities in excitation-contraction coupling, and acquired mitochondrial dysfunction are all complexly linked to weakness (2–4). It has a detrimental effect on the patient’s quality of life and capacity to resume employment (5). Additionally, it raises the cost of healthcare-related expenses and affects the rate of hospital readmissions (6). Post-ICU individualized rehabilitation is a proposed method for improving long-term wellness in ICU survivors (7). Early identification of subgroups that may benefit most from follow-up and targeted recovery programs (i.e., those with significant mid- and long-term disabilities) would be highly valuable (8).

Carnitine is required for the transport of long-chain fatty acids from the cytoplasm into the mitochondria for *β*-oxidation of fatty acid, and for the binding of acyl residues deriving from the intermediary metabolism of amino acids in order to facilitate their elimination (9). Carnitine is esterified into acylcarnitines (AC) derivatives as a result of these processes. Carnitine deficiency, particularly in the context of primary deficiency, has been associated with muscle weakness (10). In case of fatty acid *β*-oxidation is impaired and non-oxidized fatty acyl-CoA esters accumulate into the mitochondria. These esters will enter the bloodstream after being further conjugated to carnitine, primarily as long-chain AC (11).

In the 3 months after ICU discharge, a recent study found that ICU survivors had a changed serum AC profile, indicating chronic metabolic disturbances that included mitochondrial dysfunction and branched chain amino acid catabolism (12). The aim of the present observational study was to investigate the association between AC profile measured at ICU discharge (ICUD) and outcomes related to muscle weakness assessed 3 months later (M3) in survivors of a prolonged ICU stay.

## Methods

### Patients—data sources

Patients surviving a stay ≥ 7 days in our 58-bed tertiary ICU (including a 6-bed burn centre) are routinely invited to our post- intensive care follow-up. The follow-up begins in the ward, during the first 7 days following ICUD: a nurse-led face-to-face standardized visit allowed a first PICS screening. A face-to-face consultation is then scheduled at M3, addressing the main PICS domains, including physical status. A blood analysis looks at metabolic biomarkers and inflammation at each time point: our routine analysis includes measuring the acylcarnitines profile.

This analysis included all consecutive patients who were discharged from the intensive care unit (ICU) after a stay of at least 7 days between September 2020 and July 2023, provided that they attended the M3 consultation and completed the ICUD assessment. Additional exclusion criteria included zidovudine, cyclosporine, valproate, or cisplatin treatment [drugs known to influence AC levels (10, 13)], as well as a known primary carnitine deficiency or L-carnitine supplementation. Biological parameters and post-ICU clinical data were prospectively documented. Retrospectively gathered demographic and ICU stay data were taken from the medical charts (see Supplementary material).

Since the study did not alter patient care and the data were gathered anonymously, informed consent was not necessary in line with Belgian law, as approved by the University Hospital of Liege’s Ethics Committee (local reference 2020/424).

### Serum acylcarnitine profile

Serum AC concentrations (free carnitine (C0), C2-, C3-, C3DC, C4-, C4-DC-, C5-, C5:1-, C5DC, C5-OH, C6-, C6-DC-, C8-, C8:1-, C10-, C10:1-, C10:2-, C12-, C12:1-, C14-, C14:1-, C14:2-, C14-OH-, C16-, C16:1-, C16-OH, C18-, C18:1-, C18:2-, C18:1-OH-, C18:2-OH- carnitine) were determined by flow-injection analysis on a TQ5500 tandem mass spectrometer (Sciex, Framingham, MA, USA), using Neobase2 kit (PerkinElmer, Waltham, MA, USA) (14, 15). Short-chain ACs (SCACs) and long-chain ACs (LCACs) refer, respectively, to C3 + C4 + C5 and C14 + C16 + C18. An AC/C0 ratio exceeding 0.4 is attributed to a disturbed mitochondrial metabolism (10, 13).

### Muscle outcomes

Muscle mass was approximated using biomarkers. The serum urea/creatinine ratio was calculated: muscle catabolism is associated with an elevated ratio (16). The Sarcopenia Index was defined as [(serum creatinine/serum cystatin C) x 100]: a low index reflects a reduced muscle mass (17, 18). Peripheral muscle strength was determined by using handgrip and quadriceps dynamometry. The measurement method is described in the Supplementary material. Respiratory muscle strength was explored by measuring the maximum respiratory pressures at the mouth (maximal inspiratory pressure / MIP and maximal expiratory pressure / MEP) with the Micro 5,000 spirometer and the software Exp’Air (Medisoft – MGC Diagnostics, Dinant, Belgium). The measurement method is described in the Supplementary material. All measurements were performed by a team of two trained physiotherapists, using standardized protocols, thus minimized inter-rater variability.

Muscle health was defined arbitrarily for this study as a composite of urea/creatinine ratio, sarcopenia index, quadriceps and handgrip strengths, MIP and MEP. As they were expressed on different units and scales, all these data all variables were standardized (*Z*-scores) and summed to create a composite muscle health score. This pragmatic approach aimed at capturing an overall estimate of muscle status while avoiding overrepresentation of any single parameter.

### Analysis

Qualitative parameters were expressed as counts and percentages. Normality of the distribution of the quantitative variables was investigated graphically with histograms and quantile-quantile plots and assessed using the Shapiro–Wilk hypothesis test. As some datasets did not pass the normality test, results were expressed as medians with lower and upper quartiles (P25–P75) for quantitative parameters. Missing values were not replaced.

Correlations between AC profile at ICUD and the different muscle outcomes at M3 were tested using the non-parametric Spearman correlation coefficient. The association between AC profile at ICUD and muscle health at M3 was tested using a multivariate linear regression model. A STEPWISE selection based on Akaike information criterion (AIC) was performed, and the variables significantly associated with the tested outcomes were kept in the final models.

Statistical significance was defined as a *p* value less than 0.05.

## Results

From December 2020 until May 2023, 389 patients were discharged alive from ICU after a stay ≥ 7 days and benefited from the ICUD assessment, including blood sampling for AC profile characterization. Out of them, 230 patients did not attend the M3 face-to-face consultation, and 32 other patients did not complete the M3 assessment. Finally, 127 patients were assessed at both ICUD and M3 time points and were analysed (Supplementary Figure 1). Descriptive characteristics of the included subjects are detailed in Table 1. With the exception of a minor difference in age and duration of parenteral nutrition, this cohort was comparable to the patients who were not analyzed (Supplementary Table 1).

**Table 1:** Cohort demographics.

Data		*n* = 127
Age, y		63 [55–70]
Male, *n* (%)		86 (67.7)
Weight, kg		78 [66.9–92]
Height, cm		170 [164–178]
BMI, kg/m^2^		27.2 [23.1–31]
Admission category, *n* (%)	Medical	71 (55.9)
	Surgical	56 (44.1)
Primary failure, *n* (%)	Cardiovascular	43 (33.9)
	Pulmonary	30 (23.6)
	Neurologic	8 (6.3)
	Burn injury or extended wounds	8 (6.3)
	Other	38 (29.9)
SAPS II		36 [26–56]
Mechanical ventilation >24 h, *n* (%)		81 (63.8)
Duration of mechanical ventilation, d		7 [3–15]
Continuous venovenous haemofiltration, *n* (%)		9 (7)
Duration of continuous venovenous haemofiltration, d		8.5 [4.5–24.7]
Extracorporeal membrane oxygenation, *n* (%)		8 (6.3)
Propofol-based sedation, *n* (%)		94 (69.8)
Duration of propofol infusion, d		4 [2–12]
Parenteral nutrition, *n* (%)		25 (19.7)
Duration of parenteral nutrition, d		7 [3.5–12.5]
ICU LOS, d		13 [8–33]

Data are expressed as medians with lower and upper quartiles [P25-P75].

BMI, body mass index; ICU, intensive care unit; LOS, length of stay; SAPS II, Simplified Acute Physiology Score II.

The AC profile at ICUD and the muscle outcomes at M3 are detailed in Table 2.

**Table 2:** AC profile at ICU discharge and muscle outcomes at M3.

	*n* = 127
AC profile at ICUD	
C0 (μmol/L)	44.4 [33–52.2]
C0 deficiency, *n* (%)	2 (1.6)
AC/C0	0.37 [0.28–0.47]
SCACs (μmol/L)	1.2 [0.9–1.7]
LCACs (μmol/L)	0.9 [0.6–1.2]
Muscle outcomes at M3	
Urea / creatinine	38.3 [28.3–50.3]
Sarcopenia Index	72.3 [62.1–90.1]
Quadriceps strength (*N*/kg)	2.9 [2.1–3.7]
Handgrip strength (kg)	25 [19–34]
MIP (% of predicted value)	62.4 [45.8–96.7]

Data are expressed as medians with lower and upper quartiles [P25-P75].

AC, acylcarnitine; C0, free carnitine; ICUD, intensive care unit discharge; LCAC, long-chain acylcarnitine; MEP, maximal expiratory pressure; MIP, maximal inspiratory pressure; SCAC, short-chain acylcarnitine.

The Supplementary Table 2 presents the associations between serum AC profile markers at ICUD and muscle outcomes at M3. C0 was weakly associated with quadriceps strength (Rs = 0.28, *p* = 0.007) and LCACs was weakly associated with MIP (Rs = 0.23, *p* = 0.043).

The interactions between markers of the AC profile at ICUD and muscle health at M3, analysed in multivariate models including confounding factors, are described in the Supplementary Table 3. No independent associations were found AC markers (AC/C0, SCACs and LCACs) muscle health (respectively *p* = 0.166, *p* = 0.207 and *p* = 0.061), after adjustment for confounders.

## Discussion

Serum AC profiles, particularly AC/C0, SCACs, and LCACs, measured at ICU discharge in this cohort of survivors of a prolonged ICU stay, did not predict muscle atrophy and strength or muscle health as a composite outcome.

Predicting mid-term muscle impairment—and, more importantly capacity for muscle recovery—is clinically meaningful, as it could help tailor rehabilitation strategies to the individual patient’s needs. While several studies have shown that early prediction is possible during the first days following ICU admission, especially when physical impairments are considered alongside other PICS domains (19, 20), its relevance at this stage may be limited by the influence of ICU factors (environment, supports, and complications) that can modify risk (21, 22). In contrast, assessment at ICU discharge may offer a more comprehensive picture of the patient’s status and rehabilitation needs. In this context, blood biomarkers could have an added value. AC profile, despite being biologically plausible and promising, has not yet been confirmed as a reliable predictor in the present exploratory cohort. In our study, the borderline association between LCACs and muscle health (*p* = 0.061) suggests that LCACs remain an interesting candidate, given their known role in mitochondrial dysfunction. This signal should be further explored in studies with sufficient sample size to robustly assess their predictive value. It is also possible that a combination of different marker types—biomarkers of inflammation, catabolism, mitochondrial dysfunction as well as clinical parameters—will be required to more effectively predict physical function in ICU survivors (23, 24).

This study has potential limitations. First, this study is a retrospective analysis of prospectively recorded data, and the cohort size was limited, mainly due to missing data and lost to follow-up. This may have induced an unintentional selection bias. Nonetheless, our post-ICU clinic ensured a standardized follow up. Additionally, mass spectrometry was used to evaluate the AC profile, yielding the concentrations of C0 and all of its acyl-esters. The validity and originality of the present results should have been enhanced by these approaches. However, this study was exploratory in nature, and no *a priori* sample size calculation was performed due to the lack of robust preliminary data. As a result, the study may have been underpowered to detect clinically meaningful associations, and the findings should be interpreted with caution. Second, the empiric choice of a composite muscle health parameter and of its components may be open to criticism. However, in daily practice, we are forced to refer to simple clinical parameters to assess muscle health, in the absence of easily accessible mitochondrial markers. Third, the interpretation of the urea/creatinine ratio as a marker of muscle catabolism must be made with caution, as it can be influenced by hydration status, renal function, and protein intake. These confounders are likely to be less relevant in the post-ICU period, as patients have been discharged from the ICU in a clinically stable condition. Fourth, we chose not to include broader functional scores such as the Barthel Index because they incorporate other domains, including balance and cognition, that are less directly linked to our *a priori* hypothesis on carnitine metabolism and muscle status. Finally, the >0.4 AC/C₀ threshold we used as a reference has been validated in the context of inherited carnitine deficiency syndromes and not in critically ill patients. As carnitine metabolism has been rarely investigated in the ICU setting, we relied on existing data from other clinical contexts to guide our analysis. This extrapolation may limit the interpretability of our findings.

## Conclusion

In this cohort of ICU survivors, serum acylcarnitine profiles at ICU discharge did not significantly predict muscle outcomes at 3 months. While these findings do not support the use of Acs profile as a standalone predictor in this context, the borderline association observed for LCACs suggests that their potential biological relevance should not be dismissed. Larger, adequately powered studies are warranted to further investigate the role of long-chain acylcarnitines, possibly in combination with other clinical and biological markers, to improve the prediction of physical recovery after critical illness.

## Data Availability

The raw data supporting the conclusions of this article will be made available by the authors, without undue reservation.
