# Supplementary material for: Serum acylcarnitines profile at ICU discharge to predict mid-term muscle outcomes: an exploratory study
Source: Front Med (Lausanne). 2025 Oct 31;12:1622116. doi: 10.3389/fmed.2025.1622116 (PMC12615166; doi:10.3389/fmed.2025.1622116)
Supplement: Supplementary file 1 [file Data_Sheet_1.docx]

**Serum acylcarnitines profile at ICU discharge to predict mid-term muscle outcomes: a false hope.**

**SUPPLEMENTAL MATERIAL:**

**Method**

*Post-ICU follow-up*

Patients surviving a stay ≥ 7 days in our ICU are routinely invited to our post- intensive care follow-up, excepting in case of end-of-life condition, coma, known dementia, bedridden status or inability to communicate in French, the local language. During the first 7 days following ICU discharge (ICUD), a nurse-led face-to-face standardized visit allows a first PICS screening. A face-to-face consultation is then scheduled at least 3 months after ICU discharge (M3). These consultations are generally cancelled if patients are still hospitalized in an acute care facility or in an inpatient rehabilitation facility, or if they refuse the follow-up. These consultations are also standardized, addressing the main PICS domains, including physical status. At each time point, a blood analysis focuses on inflammation and metabolic biomarkers. In this context, measurement of acylcarnitine profile is part of our standard analysis.

*ICU related data*

These data included patients’ demographics and characteristics (age, sex), disease characteristics (score on the Simplified Acute Physiology Score / SAPS II at admission (D0), maximal C-reactive protein / CRP and creatine level in the blood), ICU and hospital characteristics (duration of mechanical ventilation, propofol, insulin and steroids administration, ICU length of stay / ICU LOS).

*Blood biomarkers*

Blood samples were collected through a central or peripheral venous line placed for clinical use, or through venous punction. Blood was drawn into serum gel and heparin tubes (BD Vacutainer, Becton, Dickinson and Company, Franklin Lakes, NJ, USA), before being centrifuged (3500 rpm, 15 min, 4°C). Supernatant was frozen at -20°C and stored for later analysis.

All biomarkers were generated from our laboratory (Unilab, CHU de Liège) accredited according to ISO 15.189 guidelines. The following markers were assessed: urea, enzymatic creatinine (Alinity C, Abbott, Lake Bluff, IL, USA) and cystatin C (Cobas, Roche, Mannheim, Germany).

*Serum acylcarnitines profile measurement*

Reference ranges for all measured acylcarnitines were established from serum samples of 50 non-hospitalized adults aged from 18 to 81 years, including 46% males (23/50).

*Muscle strength measurement:*

Handgrip strength was assessed using a Jamar hydraulic hand dynamometry. Measurements were performed in a sitting position, with the elbow in 90° flexion. The protocol consisted of three consecutive maximal contractions for each muscle group, preceded by three warm-up trials. Observers provided standardized encouragement. The three measurements were performed with 30-s intervals between contractions. Patients were asked to gradually increase their muscle force to a maximum effort which had to be sustained for 6-s. The highest performance was considered for analysis. Grip strength varies with age and sex: strength ≥ 25kg in men ≤ 60 years and ≥ 23kg in men between 61 and 79 years or strength ≥ 14kg in women ≤ 60 years and ≥ 13 kg in women between 61 and 79 years are considered normal [1]. Maximal isometric quadriceps strength was measured with a MicroFET2 hand-held dynamometer (Hoggan Industries, Inc., West Jordan, UT, USA), with patient lying on supine position. The highly standardized testing protocol is detailed in a previously published validation study [2]. Magnitude of the values that can be observed in healthy and critically ill patients are described elsewhere [3]. To reduce inter-individual variability and minimize the effect of subject weight on muscle strength, absolute strength was normalized according to actual body weight (expressed in N/kg). Unfortunately, at present, no cut-off values for quadriceps weakness are defined in critical care populations using the above-described protocol.

*Respiratory muscle strength measurement:*

The Micro5000 spirometer was calibrated semi-automatically before each measure using a 3-liter calibration syringe, according to manufacturer’s recommendations. The ambient conditions were used to calculate BTPS (body, temperature and pressure saturation) correction factor. The measurements were completed by patients in a seated position. The arms are in neutral position. One hand brings the pneumotachograph to the mouth and the other is placed on the patient’s thigh. A noseclip is used for all maneuvers. The mouthpiece is placed between patient’s lips and teeth in such a way as to prevent any air leakage around the mouthpiece. An antibacterial filter is placed between the mouthpiece and the device. Before the maximal inspiratory pressure (MIP)/ maximal expiratory pressure (MEP) test, a measurement of the total lung capacity, the vital capacity, the functional residual capacity and the residual volume are carried out. To measure the MIP, it was requested a maximum expiration up to the residual volume and then an inspiration against occlusion. To measure the MEP, the patient was instructed to take a maximum inspiration to reach the total lung capacity, and then to expire against occlusion. A pressure plateau of at least 1 second was required for all measures. Each test was repeated 3 times. A 60-second rest interval was applied between each trial. The highest MIP and MEP values (expressed as cmH2O) were recorded and converted into individual percentages of predicted values (% predicted) corrected for age and sex

*Statistical analysis*

Statistical analysis was performed using RStudio 2024.09.0 (Build 375).

**Supplemental Table 1: Demographics of analyzed patients compared to those discharged alive from ICU after a stay ≥ 7 days but not assessed at ICUD.**

Data are expressed as medians with lower and upper quartiles [P25-P75]. Comparisons between data were made using the Wilcoxon signed-rank test and Fisher’s exact test, as required.

*BMI: body mass index; ICU: intensive care unit, LOS: length of stay; SAPS II: Simplified Acute Physiology Score II*

| Data | | Analyzed patients (n=127) | Patients not assessed at ICUD (n=389) | p value |
| --- | --- | --- | --- | --- |
| Age, y | | 63 [55-70] | 67 [59-74] | 0.0023 |
| Male, n (%) | | 86 (67.7) | 252 (64.6) | 0.5916 |
| Weight, kg | | 78 [66.9-92] | 77 [65.8-90.7] | 0.5045 |
| Height, cm | | 170 [164-178] | 170 [163-176] | 0.5587 |
| BMI, kg/m^2^ | | 27.2 [23.1-31] | 26.8 [23.2-30.4] | 0.6575 |
| Admission category, n (%) | Medical | 71 (55.9) | 245 (63) | 0.1729 |
|  | Surgical | 56 (44.1) | 144 (37) |  |
| Primary failure, n (%) | Cardiovascular | 43 (33.9) | 102 (26.2) | <0.0001 |
|  | Pulmonary | 30 (23.6) | 61 (15.6) |  |
|  | Neurologic | 8 (6.3) | 12 (3.2) |  |
|  | Burn injury or extended wounds | 8 (6.3) | 4 (1) |  |
|  | Other | 38 (29.9) | 210 (54) |  |
| SAPS II | | 36 [26-56] | 38 [31-50] | 0.9067 |
| Mechanical ventilation >24h, n (%) | | 81 (63.8) | 256 (65.8) | 0.6694 |
| Duration of mechanical ventilation, d | | 7 [3-15] | 7 [2-14] | 0.7839 |
| Continuous venovenous haemofiltration, n (%) | | 9 (7) | 36 (9.2) | 0.5870 |
| Duration of continuous venovenous haemofiltration, d | | 8.5 [4.5-24.7] | 7.5 [7-14.2] | 0.6312 |
| Extracorporeal membrane oxygenation, n (%) | | 8 (6.3) | 12 (3.2) | 0.1146 |
| Propofol-based sedation, n (%) | | 94 (69.8) | 267 (68.6) | 0.2668 |
| Duration of propofol infusion, d | | 4 [2-12] | 5 [1.5-9] | 0.7871 |
| Parenteral nutrition, n (%) | | 25 (19.7) | 62 (16.1) | 0.3406 |
| Duration of parenteral nutrition, d | | 7 [3.5-12.5] | 6 [4-12] | <0.0001 |
| ICU LOS, d | | 13 [8-33] | 13 [10-19] | 0.9481 |

**Supplemental Table 2: Correlations between AC profile at ICUD and M3 muscle outcomes.**

Data are expressed as Spearman correlation coefficients and p value.

*AC: acylcarnitines; C0: free carnitine; HG: handgrip strength; LCAC: long-chain acylcarnitine; MEP: maximal expiratory pressure; MIP: maximal inspiratory pressure; NS: non significant; QS: quadriceps strength; SCAC: short-chain acylcarnitine; SI: sarcopenic index; UC: urea/creatinine ratio*

|  | UC | SI | QS | HG | MIP | MEP |
| --- | --- | --- | --- | --- | --- | --- |
| C0 | NS | NS | RS=0.28  P=0.007 | NS | NS | NS |
| AC/C0 | NS | NS | NS | NS | NS | NS |
| SCACs | NS | NS | NS | NS | NS | NS |
| LCACs | NS | NS | NS | NS | RS= 0.23  P=0.043 | NS |

**Supplemental Table 3: Effect of AC profile at ICUD on M3 muscle health.**

*AC: acylcarnitines; C0: free carnitine; CRP: c-reactive protein; ICU: intensive care unit; ICUD: intensive care unit discharge; LCAC: long-chain acylcarnitine; LOS: length of stay; MV: mechanical ventilation; SAPS II: Simplified Acute Physiology Score II; SCAC: short-chain acylcarnitine*

| Models including AC profile at ICUD | Variables | Beta Coefficients | Standard error | t value | p value |
| --- | --- | --- | --- | --- | --- |
| C0 | C0  ICU LOS  SAPS II  MV duration  Insulin duration  Steroids  Sex | 51.45  1.82  1.49  -4. 00  1.78  -20.72  -64.34 | 35.99  1.02  0.38  1.21  0.87  14.64  14.56 | 1.429  1.791  3.900  -3.306  2.048  -1.415  -4.418 | 0.166  0.086  0.001  0.003  0.052  0.170  0.001 |
| SCACs | SCACs  ICU LOS  SAPS II  MV duration  Max blood CRP  Max blood creatinine  Sex | -21.03  -3.58  -2.17  5.27  -0.14  22.53  46.90 | 16.21  1.64  0.98  2.35  0.09  12.98  24.08 | -1.297  -2.188  -2.496  2.241  -1.641  1.736  1.948 | 0.207  0.039  0.020  0.035  0.114  0.096  0.064 |
| LCACs | LCACs  MV duration  Propofol duration | 65.71  3.08  -4.10 | 33.64  1.64  2.24 | 1.954  1.877  -1.829 | 0.061  0.071  0.078 |

**Supplemental Figure 1: Flow chart**

**References:**

1. Spies C, Krampe H, Paul N, Denke C, Kiselev J, Piper S, Kruppa J, Grunow JJ, Steinecke K, Gülmez T *et al*: **Instruments to measure outcomes of post-intensive care syndrome in outpatient care settings – Results of an expert consensus and feasibility field test**. *Journal of the Intensice Care Society* 2020.

2. Rousseau AF, Kellens I, Freycenon G, Dardenne N, Bruyere O, Damas P, Croisier JL: **Highly standardized quadriceps dynamometry of critically ill adults at bedside: a step towards individualized rehabilitation.** *Acta Anaesthesiol Belg* 2018, **69**(3):159-164.

3. Blanjean A, Kellens I, Misset B, Joris J, Croisier JL, Rousseau AF: **Quadriceps strength in intensive care unit survivors: Variability and influence of preadmission physical activity**. *Aust Crit Care* 2020.
